# Role of fluorine in two-dimensional dichalcogenide of SnSe_**2**_

**DOI:** 10.1038/s41598-018-20111-y

**Published:** 2018-01-26

**Authors:** Jin Tae Kim, Da Seul Hyeon, Kota Hanzawa, Ayaka Kanai, Se Yun Kim, Yong Jei Lee, Hideo Hosono, Joonho Bang, Kimoon Lee

**Affiliations:** 1 Department of Physics, Kunsan National University, Gunsan, 54150 Republic of Korea; 2 Materials and Structures Laboratory, Tokyo Institute of Technology, Yokohama, 226-8503 Japan; 3 Materials R&D Center, Samsung Advanced Institute of Technology, Suwon, 16678 Republic of Korea; 4 Materials Research Center for Element Strategy, Tokyo Institute of Technology, Yokohama, 226-8503 Japan

## Abstract

Authors report an effect of F substitution on layered SnSe_2_ through the successful synthesis of polycrystalline SnSe_2***−****δ*_F_*x*_ (0.000 ≤ *x* ≤ 0.010) by solid-state reaction. Accompanied with density functional theory calculations, the blue shift of A_1g_ peak in Raman spectra reveal that F^−^ ions are substituted at Se vacancy sites as decreasing the reduced mass of vibrational mode associated with Sn–Se bonding. From the measurements of electrical parameters, conductivity as well as carrier concentration are governed by thermally activated behavior, while such behavior is suppressed in Hall mobility, which occurs as F ratio increases. Based on Arrhenius relation, it is found that the potential barrier height at the grain boundary is suppressed with increasing F amount, suggesting that the F^−^ ion is a promising candidate for the grain boundary passivation in the two-dimensional dichalcogenide system.

## Introduction

Two-dimensional (2D) transition metal dichalcogenides (TMDs) have recently attracted much attention from researchers due to its novel electronic and/or optical properties^1–5^. Since the isolation of few-layered MoS_2_ has successfully triggered ballistic transport behavior^3^, various kinds of TMDs between other transition metals and chalcogen elements have also been investigated to explore distinctive physics, such as valley-related transport or Weyl semi-metallic state^4,5^. However, most of the studies have mainly been conducted on limited transition metal cation-based composition (Mo, W, and Re, etc.), although there are many other groups of layered dichalcogenides containing weak van der Waals bonding between layer units^1–5^.

Among such layered materials, SnSe_2_, post-transition metal dichalcogenide (PTMD), is regarded as a promising electronic material^6–13^. Yu *et al*. reported that bi-layered SnSe_2_ field-effect transistor shows relatively fast photoresponse at room temperature with a high photo-to-dark ratio^11^. Notably, unlike in transition metal cation-based TMDs, electrical resistivity of SnSe_2_ can be easily controlled by conventional chemical substitution, as we have reported previously^12^. Accompanied with the highly dispersive conduction path derived from Sn s-orbital, stable Cl substitution on Se-site was attained as resulting in metallic conduction under a high electron carrier concentration up to ~10^20^ cm^−3^. Considering that it has been a challenge to obtain such a high carrier concentration using chemical substitution in semiconducting 2D materials, the anion substitution method opens up a new approach for engineering physical properties of 2D materials. Theoretical studies suggest that anion substitution using other halogen elements such as F and Br can be also effective for the electron doping in SnSe_2_^13^; however, no experimental investigation on these substitutions has been reported.

In this paper, we report the effects of F^−^ ion in 2D SnSe_2_ material. By solid-state reaction, polycrystalline SnSe_2−*δ*_F_*x*_ (*δ* indicates selenium vacancies^14,15^) are successfully synthesized with various nominal F contents up to *x* = 0.010. From the Raman spectra, peak shift of the characteristic A_1g_ mode is observed as verifying the substitution of F^−^ ion on Se-site. Density functional theory (DFT) calculations well support that the substitution of F in the form of F^−^ is more stable than the formation of a selenium vacancy at the Se site in SnSe_2_. By introducing the F in SnSe_2_, the potential barrier height is monotonically decreased at the grain boundaries where the Se anion is relatively deficient compared to the intra-grain region. It strongly suggests that the grain boundary passivation can be achieved by F^−^ ions which is analogous to the hydrogen passivation in the polycrystalline silicon^16,17^.

## Results and Discussion

**Figure 1:**
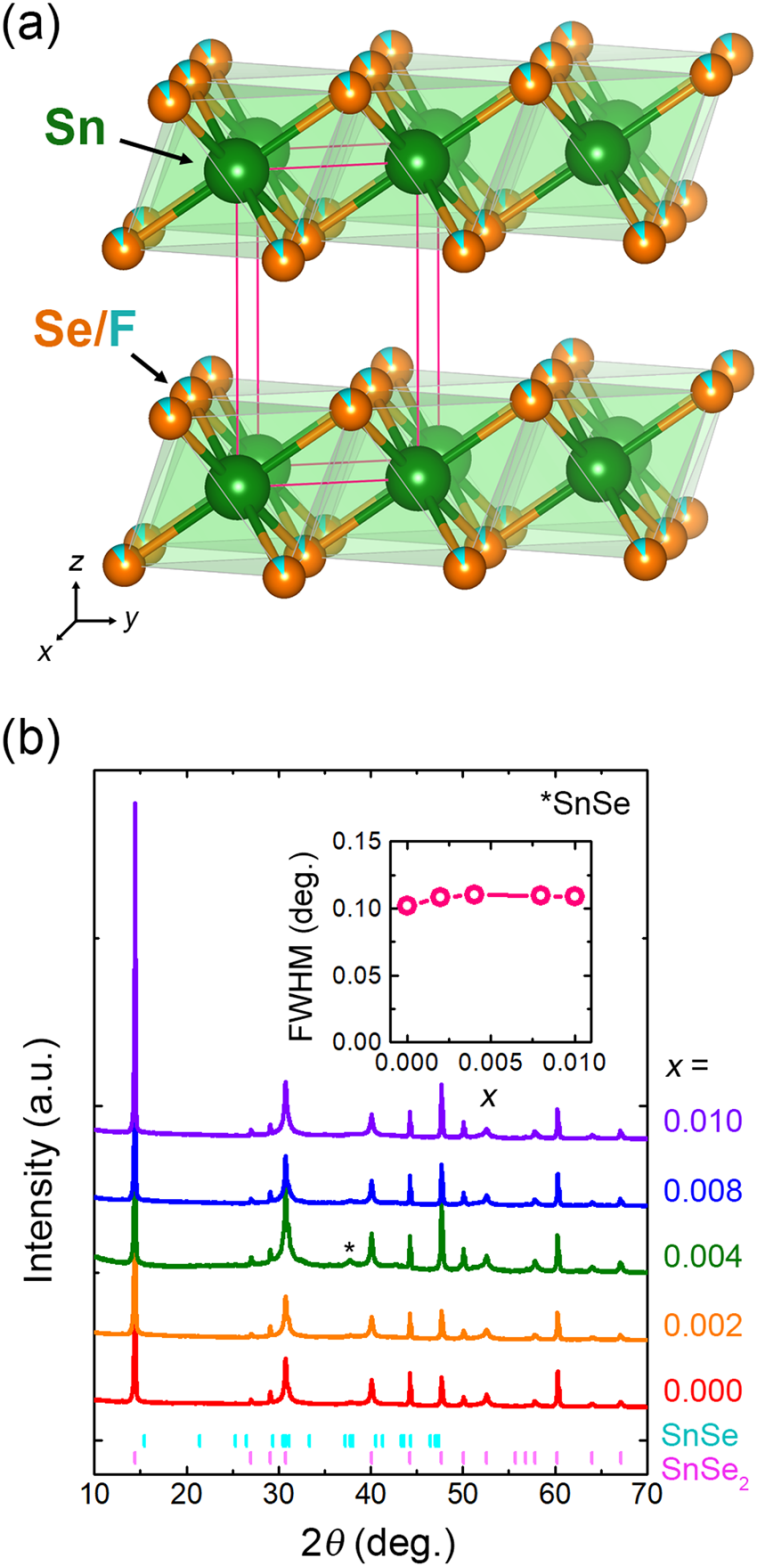
Structural data obtained from the PXRD measurements. (**a)** Crystal structure of the F-incorporated SnSe_2_. (**b**) PXRD patterns of the SnSe_2−*δ*_F_*x*_ samples with various F contents. Asterisk (*) indicates the peak associated with a secondary phase of SnSe. Inset shows the *x* dependent full-width at half-maximum (FWHM) of (001) peak.

**Figure 2:**
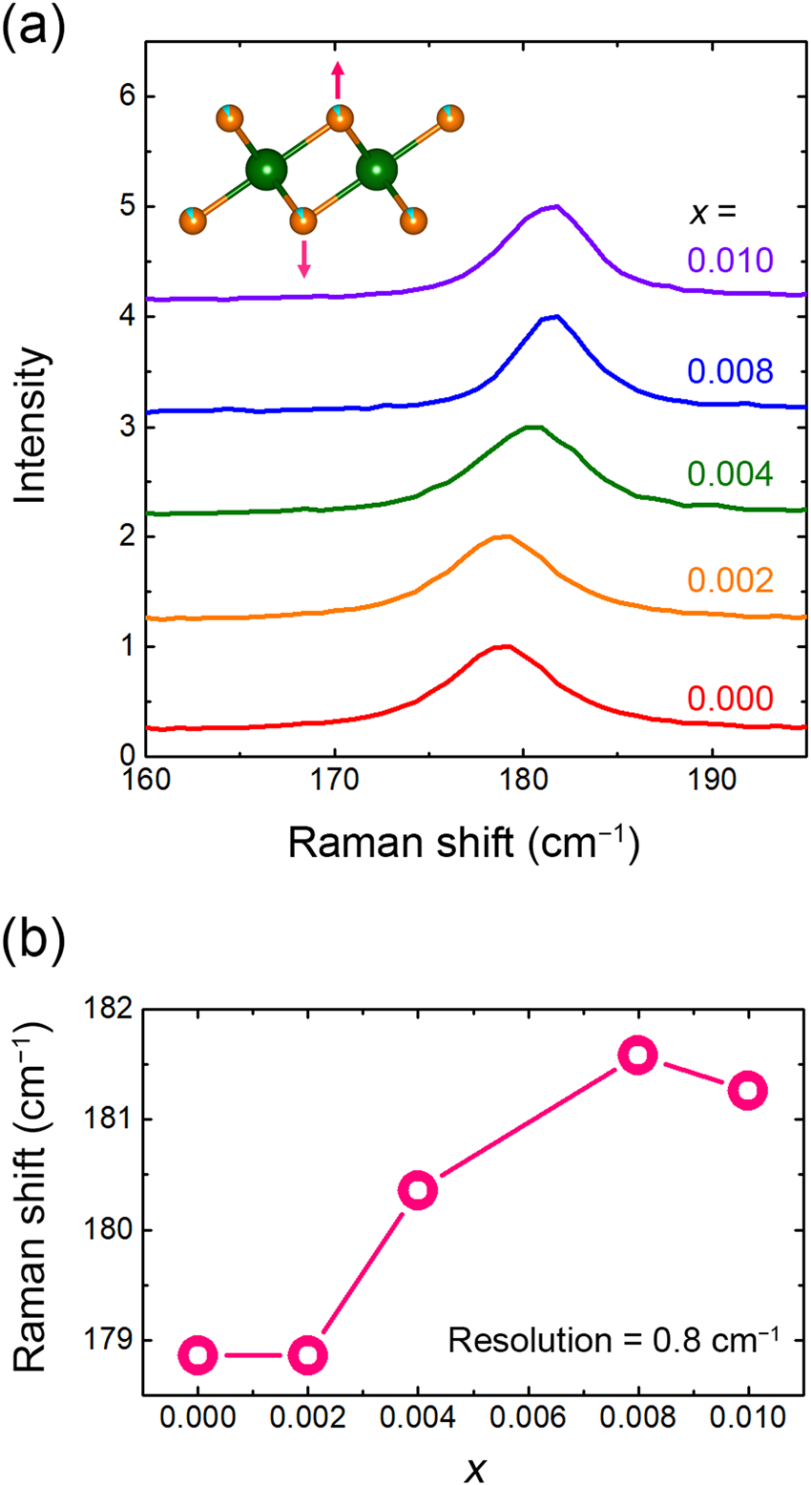
Vibrational properties of the SnSe_2−*δ*_F_*x*_ samples with various F contents. (**a**) Raman spectra of the F-incorporated SnSe_2_ obtained at room temperature. (**b)** The peak position of A_1g_ mode with the different F content.

Figure 1a and b show the schematic of crystal structure and powder X-ray diffraction (PXRD) patterns, respectively, for SnSe_2−*δ*_F_*x*_ samples with various F contents. All samples show the hexagonal layered-structure (space group: *P*-3*m*1, see  Figure 1a) with a small amount of secondary-phase SnSe (indicated by an asterisk). There is no meaningful change in the lattice constants for all *x*, suggesting that F dopants do not occupy the inter-layer sites as intercalants, but occupy the substitutional sites to minimize the lattice deformation, as reported previously from the case of Cl substitution^12^. Raman spectra as shown in Figure 2a strongly support these aspects. As shown in Figure 2a, all samples exhibit the peak around 180 cm^−1^, which is associated with the vibrational mode of the Sn–Se bond (A_1g_)^12,18^. The F-content-dependent peak position of the A_1g_ vibrational mode is summarized in Figure 2b. Upon increasing *x*, positive shift of the A_1g_ peak is observed. From the fact that the vibrational energy is proportional to $\sqrt{k/m}$, where *k* and *m* are the spring constant and the reduced mass, respectively, the blue shift of A_1g_ peak is ascribed to the decrease of the reduced mass as forming the Sn–F bonds with partial replacement of the Se^2−^ site by lighter F^−^ ions^19,20^.

**Figure 3:**
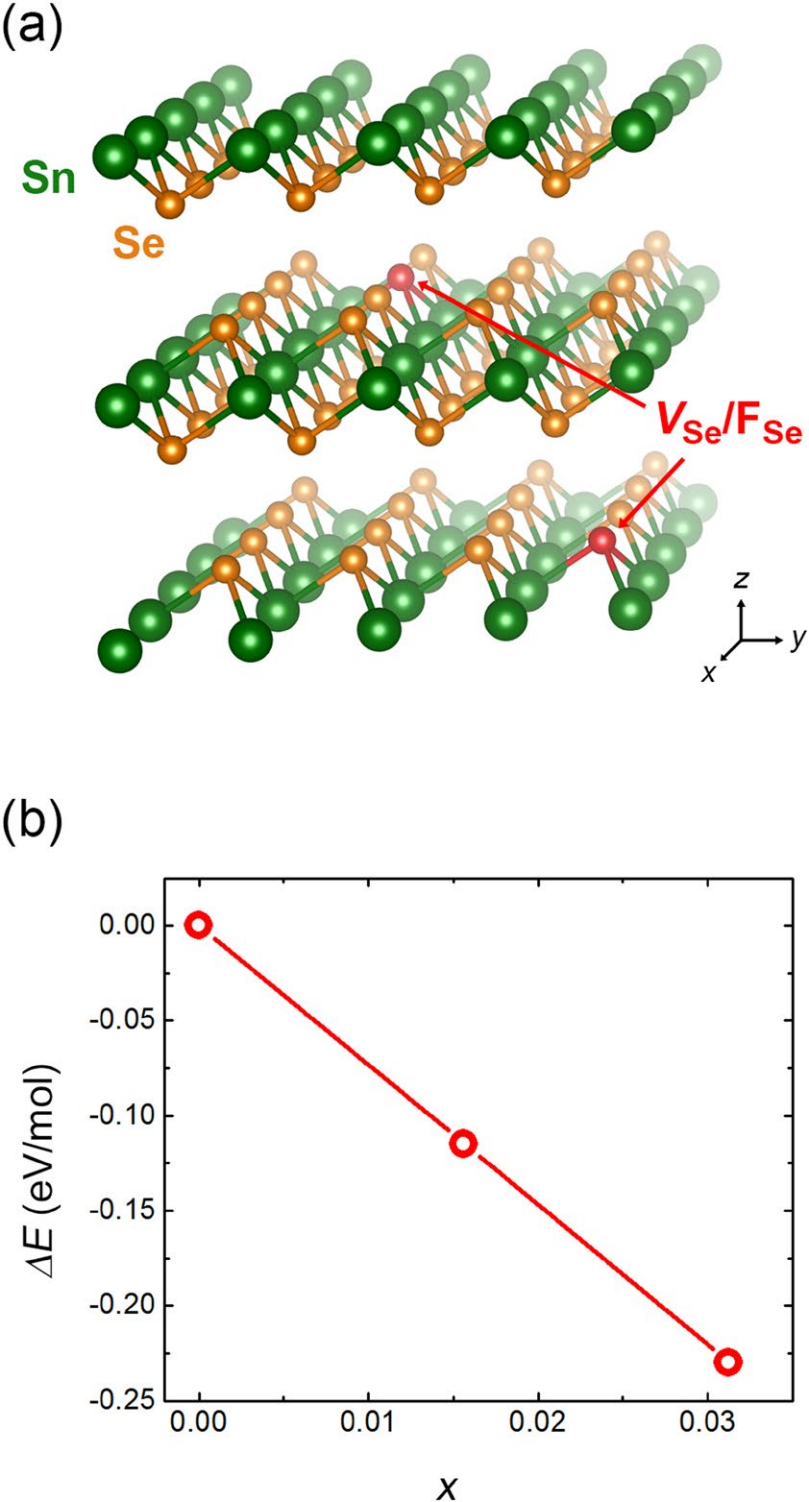
Thermodynamic stability of the F substitution on Se-site with various Se deficiency ratios. (**a**) 4 × 4 × 2 supercell structure of SnSe_2_ containing Se vacancy (*V*_Se_) or F substitution (*F*_Se_). (**b)** Calculated energy differences (Δ*E*) corresponding to the chemical reaction (1) for *x* = 0, 1/64, and 2/64.

To more firmly demonstrate the thermodynamic stability of the F substitution at the Se site in SnSe_2_, DFT calculations are performed under various Se deficient conditions. Figure 3a shows the 4 × 4 × 2 supercell structure containing 96 host atoms (32 Sn and 64 Se atoms) which is constructed for the calculations. By the partial subtraction of the Se atoms, we construct the Se deficient models for *x* = 1/64 and 2/64, respectively. The stability of the F substitution can be evaluated as the energy difference (Δ*E*) between the chemical states of the reaction formulas (1) as follows:

1 $${{\rm{SnSe}}}_{2-x}+0.5x{{\rm{F}}}_{2}\to {{\rm{SnSe}}}_{2-x}{{\rm{F}}}_{x}$$

i.e., Δ*E* = *E*(SnSe_2−*x*_F_*x*_) − [*E*(SnSe_2−*x*_) + *E*(0.5*x*F_2_)]. As displayed in Figure 3b, the calculated Δ*E* value becomes more negative as *x* increases, indicating the substitution of F in the form of F^−^ is much more favorable than the formation of a Se vacancy at the Se site when the Se becomes more deficient. Considering that the grain boundary in 2D materials contains much more anion deficiencies compared to the intra-grain regions^21,22^, it suggests that F substitution could occur more favorably at grain boundary region where Se vacancies are more concentrated (More experimental evidences will be discussed below).

**Figure 4:**
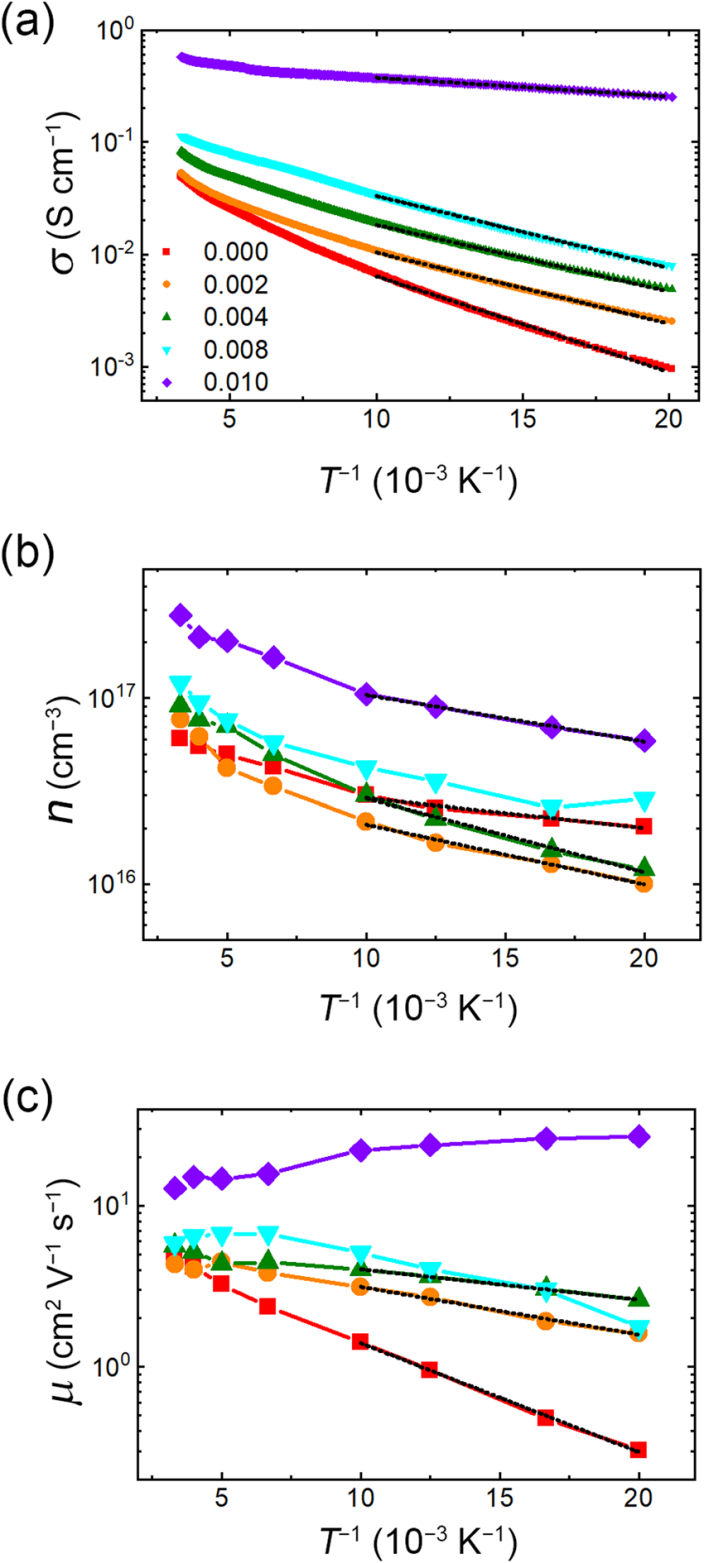
Temperature dependent electrical properties (filled symbols) and their Arrhenius fits (black dashed lines). (**a)** The electrical conductivity (*σ*), (**b**) the carrier concentration (*n*) and (**c)** the electron mobility (*μ*) with various F contents.

Figure 4a displays the temperature (*T*)-dependent electrical conductivity (*σ*) for the various F contents. The *σ* gradually increases with increasing *x* from 0.05 to 0.57 S/cm at 300 K. All samples exhibit thermally activated behavior regardless of F content amount, which differs from the case of metal–insulator transition in the Cl-substituted SnSe_2_^12^. All samples show the negative Hall coefficients indicating *n*-type character, and their carrier concentrations (*n*) are estimated as ~6 × 10^16^ (*x* = 0.000) to ~3 × 10^17^ cm^−3^ (*x* = 0.010) at 300 K (Figure 4b). Although carrier concentration is slightly enhanced with increasing *x*, it is much inferior to that from the Cl-substituted SnSe_2_^12^. This indicates that F^−^ ion is a relatively inefficient electron donor compared to Cl^−^ ion, which may be due to its deeper donor energy level as predicted by theoretical study^13^. From the relation of *σ* = *neμ*, where *e* and *μ* are the elementary charge and the electron mobility, respectively, *μ* values can be obtained as depicted in Figure 4c. It should be noted that the thermally activated behavior in *μ* is gradually suppressed by introducing F^−^ ions, and *T* dependence of *μ* finally exhibit phonon-limited scattering behavior when *x* = 0.01. Distinct from the phonon-limited scattering in *x* = 0.01, other factors should be taken into account as the dominant scattering mechanism for *T* dependence on *μ* from *x* = 0 to 0.008, such as the ionized impurity and/or grain boundary scattering which should be suppressed as *T* increased^23,24^. Because the ionized impurity scattering should be increased with increasing amount of impurity (F^−^ ions), the suppression of thermally activated behavior in *μ* mainly originates from the depressed grain boundary scattering as F^−^ introduced in SnSe_2_. From these results, it can be inferred that F^−^ ions should behave as a defect healer at a grain boundary, which is analogous to the passivation of the polycrystalline silicon with hydrogen^16,17^.

**Figure 5:**
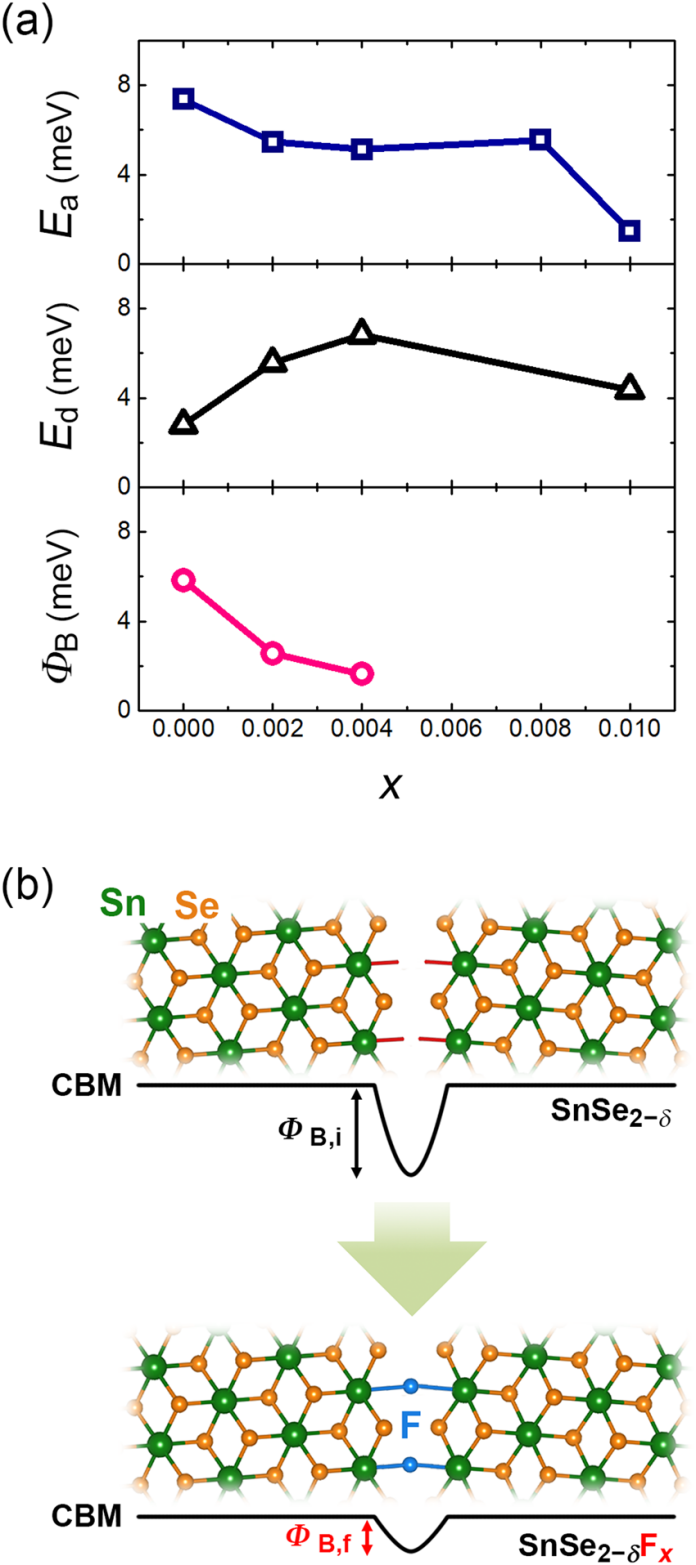
Role of the F^−^ ions in the SnSe_2_. (**a)** The activation energy (*E*_a_), the donor ionization energy (*E*_d_), and the grain-boundary height (*Φ*_B_) obtained by the Arrhenius relationship (see black dashed lines in Figure 4). (**b**) Schematic illustrations of the grain boundary passivation in the F-incorporated SnSe_2_. *Φ*_B,i_ and *Φ*_B,f_ indicate the grain boundary height for the F-free SnSe_2_ and the F-incorporated SnSe_2_, respectively. CBM means conduction band minimum.

To confirm such aspects, we quantitatively analyze *σ*, *n*, and *μ* based on the Arrhenius relation. Figure 5a shows the activation energy (*E*_a_), the donor ionization energy (*E*_d_), and the grain-boundary height (*Φ*_B_) estimated by Arrhenius relationships as follows^23–25^:

2 $$\sigma (T) \sim \exp (-\frac{{E}_{{\rm{a}}}}{kT})$$

3 $$n(T) \sim \exp (-\frac{{E}_{{\rm{d}}}}{2kT})$$

4 $$\mu (T) \sim \exp (-\frac{{\Phi }_{{\rm{B}}}}{kT})$$

where *k* is the Boltzmann constant. As *x* increases, *E*_*a*_ and *Φ*_B_ are gradually decreased, while *E*_d_ slightly increases with *x* values except for *x* = 0.01. Because the average grain size does not vary with different F contents, as estimated by full-width at half-maximum (FWHM) values for (001) peak (see the inset of  Figure 1a), the decrease of *Φ*_B_ suggests that the defects at the grain boundary, such as Se vacancies, are passivated by F^−^ ions (Figure 5b), which suppresses the grain boundary scattering^14,26^. It is worthwhile to note that DFT calculations by Huang *et al*. suggested that the substituted F at the Se-site forms the deeper energy level compared to other halogen elements^13^. If F substitution occurs in the whole region of SnSe_2_, *E*_*a*_ value should be governed by *E*_*d*_ as resulting in the decrease of *σ*, but the resultant *E*_*a*_ is mainly dominated by *Φ*_B_ rather than *E*_*d*_. This strongly supports that F dopant cannot exist as a trap center in the intra-grain region, but effectively lowers *Φ*_*B*_ as dominantly substituting on Se deficient region where dangling bond exists^21,22^, (see Figure 5b) resulting in decrease on *E*_*a*_ as well as *σ* enhancement.

## Conclusions

In summary, the effects of F^−^ ions in SnSe_2_ are investigated. Polycrystalline SnSe_2−*δ*_F_*x*_ with various nominal F contents are synthesized by solid-state reaction. Along with structural analysis and DFT calculations, Raman spectra verify that F^−^ ions well substitute on Se-sites, resulting in the blue shift of A_1g_ peak, which is associated with vibrational mode of Sn–Se bonding. The *T*-dependent *σ* and *n* are dominated by thermally activated behavior, but such behavior is effectively suppressed in *μ* with increasing F contents. Based on Arrhenius relationship, we can conclude that the substitution of F^−^ ions mainly occurs at the grain boundaries, thus successfully lowering the grain barrier height rather than acting as a shallow electron donor. The present study suggests that the F^−^ ion is a promising candidate for the grain boundary passivation in the 2D dichalcogenide system.

## Methods

### Sample synthesis

Polycrystalline SnSe_2−*δ*_F_*x*_ (0.000 ≤ *x* ≤ 0.010) in the form of sintered pellets were synthesized by solid-state reaction. Stoichiometric amounts of Sn, Se and anhydrous SnF_2_ powders were mixed:

$$(2-x)\,{\rm{S}}{\rm{n}}+{x}{\rm{S}}{\rm{n}}{{\rm{F}}}_{2}+2(2-x){\rm{S}}{\rm{e}}\to 2{\rm{S}}{\rm{n}}{\rm{S}}{{\rm{e}}}_{2-x}{{\rm{F}}}_{{x}}.$$

Mixed precursors were sealed under vacuum in the silica tubes. The reaction was performed by two-step process: the mixed precursors were heated at 400 °C for 48 hours and followed by heating of the pelletized sample at 500 °C for another 48 hours with additional grinding. To prevent the vaporization of the F, samples were slowly heated at 10 °C/hour.

### Structural and electrical characterizations

Crystal structure was characterized by PXRD and Raman spectroscopy at room temperature [Empyrean (PANaytical) and NTEGRA (NT-MDT), respectively]. The temperature-dependent electrical properties were measured from 50 to 300 K using a physical property measurement system (Quantum Design). To measure the electrical properties, we fabricated a four point probe and Hall bar configuration on the samples by applying Ag paste electrodes. The dimension of samples is 1 × 0.5 × 0.1 cm^3^ (length × width × thickness), and applied electric current is 5 mA for each measurements.

### DFT calculations

DFT calculations were performed using the generalized gradient approximation with the Perder–Burke–Ernzerhof functional and the projector augmented plane-wave method implemented in the Vienna *ab initio* simulation program code^27–29^. Self-consistency was carried out using a 4*a* × 4*b* × 2*c* supercell containing 96 atoms, and a 3 × 3 × 3 *k*-point mesh was used. The plane-wave basis set cut-off energy was set to 550 eV and the structural relaxations were performed until the Hellmann–Feynman forces were less than 10^−3^ eV Å^−1^.
